# Ubiquitin-specific protease 14 regulates c-Jun N-terminal kinase signaling at the neuromuscular junction

**DOI:** 10.1186/1750-1326-10-3

**Published:** 2015-01-10

**Authors:** Jada H Vaden, Bula J Bhattacharyya, Ping-Chung Chen, Jennifer A Watson, Andrea G Marshall, Scott E Phillips, Julie A Wilson, Gwendalyn D King, Richard J Miller, Scott M Wilson

**Affiliations:** Department of Neurobiology, University of Alabama at Birmingham, Civitan International Research Center, Evelyn F. McKnight Brain Institute, 1825 University Blvd, Birmingham, AL 35294 USA; Department of Molecular Pharmacology and Biological Chemistry, Northwestern University, 303 East Chicago Ave, Chicago, IL 60611 USA; Department of Structural Biology, St. Jude Children’s Research Hospital, Danny Thomas Place, Memphis, TN 38105-3678 USA; Department of Physiology, Neurobiology and Behavior, University of California, Davis, California, CA USA

**Keywords:** USP14, JNK, Ubiquitin, Neuromuscular junction, MLK3, Synapse, Ubiquitin proteasome system, K63-linked ubiquitin, Motor neuron, Motor endplate disease

## Abstract

**Background:**

Ubiquitin-specific protease 14 (USP14) is one of three proteasome-associated deubiquitinating enzymes that remove ubiquitin from proteasomal substrates prior to their degradation. *In vitro* evidence suggests that inhibiting USP14’s catalytic activity alters the turnover of ubiquitinated proteins by the proteasome, although whether protein degradation is accelerated or delayed seems to be cell-type and substrate specific. For example, combined inhibition of USP14 and the proteasomal deubiquitinating enzyme UCH37 halts protein degradation and promotes apoptosis in multiple myeloma cells, whereas USP14 inhibition alone accelerates the degradation of aggregate-prone proteins in immortalized cell lines. These findings have prompted interest in USP14 as a therapeutic target both inside and outside of the nervous system. However, loss of USP14 in the spontaneously occurring *ataxia* mouse mutant leads to a dramatic neuromuscular phenotype and early perinatal lethality, suggesting that USP14 inhibition may have adverse consequences in the nervous system. We therefore expressed a catalytically inactive USP14 mutant in the mouse nervous system to determine whether USP14’s catalytic activity is required for neuromuscular junction (NMJ) structure and function.

**Results:**

Mice expressing catalytically inactive USP14 in the nervous system exhibited motor deficits, altered NMJ structure, and synaptic transmission deficits that were similar to what is observed in the USP14-deficient *ataxia* mice. Acute pharmacological inhibition of USP14 in wild type mice also reduced NMJ synaptic transmission. However, there was no evidence of altered proteasome activity when USP14 was inhibited either genetically or pharmacologically. Instead, these manipulations increased the levels of non-proteasome targeting ubiquitin conjugates. Specifically, we observed enhanced proteasome-independent ubiquitination of mixed lineage kinase 3 (MLK3). Consistent with the direct activation of MLK3 by ubiquitination, we also observed increased activation of its downstrea targets MAP kinase kinase 4 (MKK4) and c-Jun N-terminal kinase (JNK). *In vivo* inhibition of JNK improved motor function and synapse structure in the USP14 catalytic mutant mice.

**Conclusions:**

USP14’s catalytic activity is required for nervous system structure and function and has an ongoing role in NMJ synaptic transmission. By regulating the ubiquitination status of protein kinases, USP14 can coordinate the activity of intracellular signaling pathways that control the development and activity of the NMJ.

**Electronic supplementary material:**

The online version of this article (doi:10.1186/1750-1326-10-3) contains supplementary material, which is available to authorized users.

## Background

Protein ubiquitination is an exceptionally flexible post-translational modification because ubiquitin can be conjugated onto substrates in different lengths and linkages, enabling it to regulate a wide variety of signaling pathways [1, 2]. For example, lysine 63 (K63)- linked chains regulate the endocytosis and sorting of plasma membrane receptors. [3]. There is also an emerging link between K63-linked ubiquitination and kinase activation [4, 5], with K63-linked chains serving as a scaffold for the recruitment of signaling components or, in the case of MLK3, directly inducing dimerization and kinase activation [6]. In contrast, K48-linked ubiquitin chains target proteins for proteasomal degradation.

USP14 is one of three proteasome-associated deubiquitinating enzymes (DUBs) that remove ubiquitin from proteasomal substrates prior to their degradation, thus terminating the ubiquitin signal and maintaining a stable pool of free ubiquitin [7–9]. In addition, USP14’s catalytic activity may alter the turnover of ubiquitinated proteins by the proteasome in a cell-type and substrate specific manner [10–13]. Combined inhibition of USP14 and the proteasomal DUB ubiquitin C-terminal hydrolase 37 (UCH37) in multiple myeloma cells delays proteasome-mediated protein degradation, halts the cell cycle, and leads to tumor cell apoptosis [14]. In contrast, in immortalized cell lines, inhibition of USP14 appears to accelerate the degradation of proteins known to aggregate in neurological diseases [11]. Additional proteolysis-independent functions for USP14 have also been described, including deconjugating K63-linked ubiquitin chains on Disheveled 2 [15] and controlling the cell-surface expression of GABAA receptors [16].

Despite the growing interest in USP14 inhibition as a treatment for neurological diseases ([11, 17] but see [18]) and cancers [13, 14, 19, 20], loss of USP14 in the spontaneously occurring *ataxia* (*ax*^*J*^) mouse mutant causes a severe loss of mobility and early postnatal lethality [21]. The neuromuscular phenotype of the *ax*^*J*^ mice is rescued by neuronal-specific expression of USP14 [22], demonstrating a critical need for USP14 in the nervous system. In this study, we used genetic and pharmacological inhibition of USP14 to investigate the contributions of USP14’s catalytic activity to NMJ structure and function. Expression of a catalytically inactive form of USP14 in the nervous system caused developmental deficits in NMJ structure and synaptic transmission. However, acute pharmacological inhibition of USP14 at adult NMJs also significantly reduced synaptic transmission, indicating that USP14 participates in dynamic ubiquitin signaling events that support neurotransmitter release. This ubiquitin signaling appears to be independent of proteasomal-mediated protein degradation. Instead, our data suggest that USP14 disassembles non-proteasomal-targeting ubiquitin chains and indicate that loss of USP14’s DUB activity leads to enhanced K63-linked ubiquitination of MLK3 and hyperactivation of its signaling cascade. Inhibition of pJNK, which is downstream of MLK3, significantly improved the motor deficits and NMJ pathology caused by loss of USP14’s DUB activity. These findings demonstrate that USP14 is involved in regulating multiple ubiquitin signals in the nervous system, ranging from acute ubiquitination for the maintenance of synaptic activity to long-term control of ubiquitin pools.

## Results

### Tg*Usp14CA*displaces endogenous USP14 from the proteasome without altering proteasome activity

**Figure 1:**
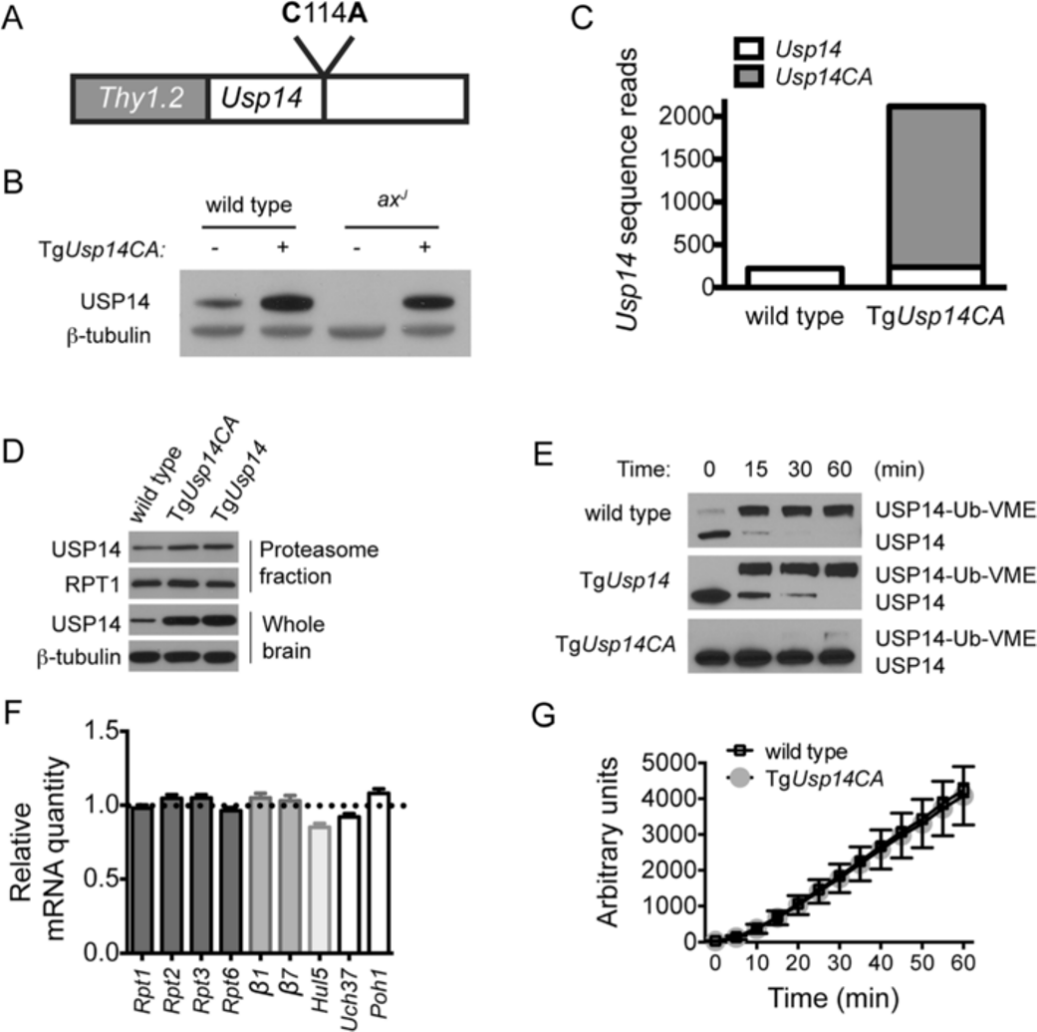
**Transgenic expression of USP14CA in the nervous system. (A)** Full length *Usp14* cDNA containing a mutation of the active site cysteine to alanine (C114A) was cloned behind the *Thy1.2* promoter. **(B)** Representative immunoblot of spinal cord extracts taken from 4- to 6- week-old wild type and *ax* ^*J*^ mice (± Tg*Usp14CA*) mice using USP14-specific antisera. β-tubulin was included as a loading control. **(C)** Abundance of wild type *Usp14* and transgenic *Usp14CA* transcripts obtained by RNA transcriptome analysis of brain tissue taken from 4- to 6-week-old wild type and Tg*Usp14CA* mice. **(D)** Representative immunoblots of USP14 in whole brain lysates and brain proteasome fractions from 4- to 6-week-old wild type, Tg*Usp14CA*, and Tg*Usp14* mice. β-tubulin was included as a loading control for whole brain and RPT1 was a loading control for the proteasome fraction. Tg*Usp14* mice express wild type USP14 behind the *Thy1.2* promoter. **(E)** Ubiquitin vinyl methyl ester (Ub-VME) assay for DUB activity in proteasome fractions from the brains of 4- to 6-week-old wild type, Tg*Usp14*, and Tg*Usp14CA* mice**.** Ubiquitin is covalently attached to the active site cysteine of USP14, resulting in a slower migrating USP14 species denoted as USP14-Ub-VME. The mutant USP14CA cannot be labeled by Ub-VME. **(F)** Relative mRNA quantity of proteasome subunits and associated factors determined by qPCR, normalized to wild type (represented by dotted line on graph), from spinal cords of 6-week-old wild type and Tg*Usp14CA* mice. n = 3 mice per genotype, run in triplicate. Data are shown as mean ± SEM **(G)** Assay of trypsin-like activity of proteasomes isolated from 4- to 6-week-old wild type and Tg*Usp14CA* mice using the fluorogenic Boc-LRR-AMC substrate. n = 3 mice per genotype, and data are shown as mean ± SEM. See also Additional file 1.

To investigate the contributions of USP14’s DUB activity to nervous system structure and function, we generated transgenic mice expressing a catalytically inactive form of USP14 in the nervous system. The coding sequence for USP14’s active site cysteine was changed to an alanine residue using PCR-site-directed mutagenesis, and the resulting *Usp14C114A* cDNA was cloned behind the neuronal *Thy1.2* promoter (Figure 1A). Expression of this transgene, referred to as Tg*Usp14CA*, caused a robust increase in USP14 expression in the spinal cords of both wild type and USP14-deficient *ax*^*J*^ mice (Figure 1B) that was easily detectible by postnatal day (P) 8 (see Additional file 1). Because reduced ubiquitin levels [23] and altered NMJ structure [24] are observed in *ax*^*J*^ mice prior to P8, we chose to study the effects of Tg*Usp14CA* in wild type mice. Importantly, using RNA transcriptome analysis, we determined that Tg*Usp14CA* did not alter the expression of endogenous *Usp14* mRNA in the brains of wild type mice, and that transgenic *Usp14CA* mRNA accounted for 90% of total *Usp14* transcripts (Figure 1C). We therefore interpreted the robust increase in USP14 abundance observed in the brains and spinal cords of wild type mice expressing Tg*Usp14CA* (henceforth Tg*Usp14CA* mice) as evidence of USP14CA expression (see Additional file 1). In contrast, USP14 abundance was not increased in non-neuronal tissues of Tg*Usp14CA* mice, indicating that USP14CA was expressed exclusively in the nervous system (Additional file 1). Overall, the localization and abundance of USP14CA were consistent with what we have previously observed when wild type USP14 is expressed under the *Thy1.2* promoter [22].

While USP14 can be found both free and bound to the proteasome, its catalytic activity has only been observed when associated with the proteasome [22]. We found that expression of Tg*Usp14CA* caused increased USP14 abundance in proteasome fractions isolated from the brains of 4- to 6-week old mice that was similar to what was observed when wild type USP14 was overexpressed in the Tg*Usp14* mice (Figure 1D). To determine whether USP14CA was the predominant USP14 species on Tg*Usp14CA* proteasomes, we used a ubiquitin vinyl-methyl-ester (Ub-VME) assay [25]. Ub-VME labels catalytically active USP14 by forming a covalent bond with its active site cysteine residue, creating a shift in molecular weight that can be detected by immunoblotting. Because the active site cysteine is mutated to alanine in USP14CA, it cannot be labeled by Ub-VME. As expected, when proteasome fractions isolated from the brains of wild type and Tg*Usp14* mice were incubated with Ub-VME, all USP14 was labeled within 1 hr (Figure 1E). In contrast, only minimal USP14 labeling was observed in proteasomes isolated from Tg*Usp14CA* mice, demonstrating that USP14CA displaces endogenous USP14 from the proteasome.

Because inhibition of Upb6, the yeast ortholog of USP14, delays the degradation of ubiquitinated proteins by the proteasome and induces increased transcription of proteasomal subunits [10], we looked for signs of proteasome dysfunction in the nervous systems of Tg*Usp14CA* mice. In contrast to what is observed in yeast, we did not observe transcriptional upregulation of components of the proteasome’s catalytic core or regulatory particle in the brains of Tg*Usp14CA* mice (Figure 1F). Similarly, when the chymotrypsin-like activity of proteasomes isolated from Tg*Usp14CA* mice was compared to that of wild type proteasomes, the activity profile was identical (Figure 1G). Therefore, expression of Tg*Usp14CA* almost completely eliminated the DUB activity of USP14 on the proteasome, but did not cause proteasome stress.

### Tg*Usp14CA*reduces muscle mass, strength, and coordination but does not cause early postnatal lethality

**Figure 2:**
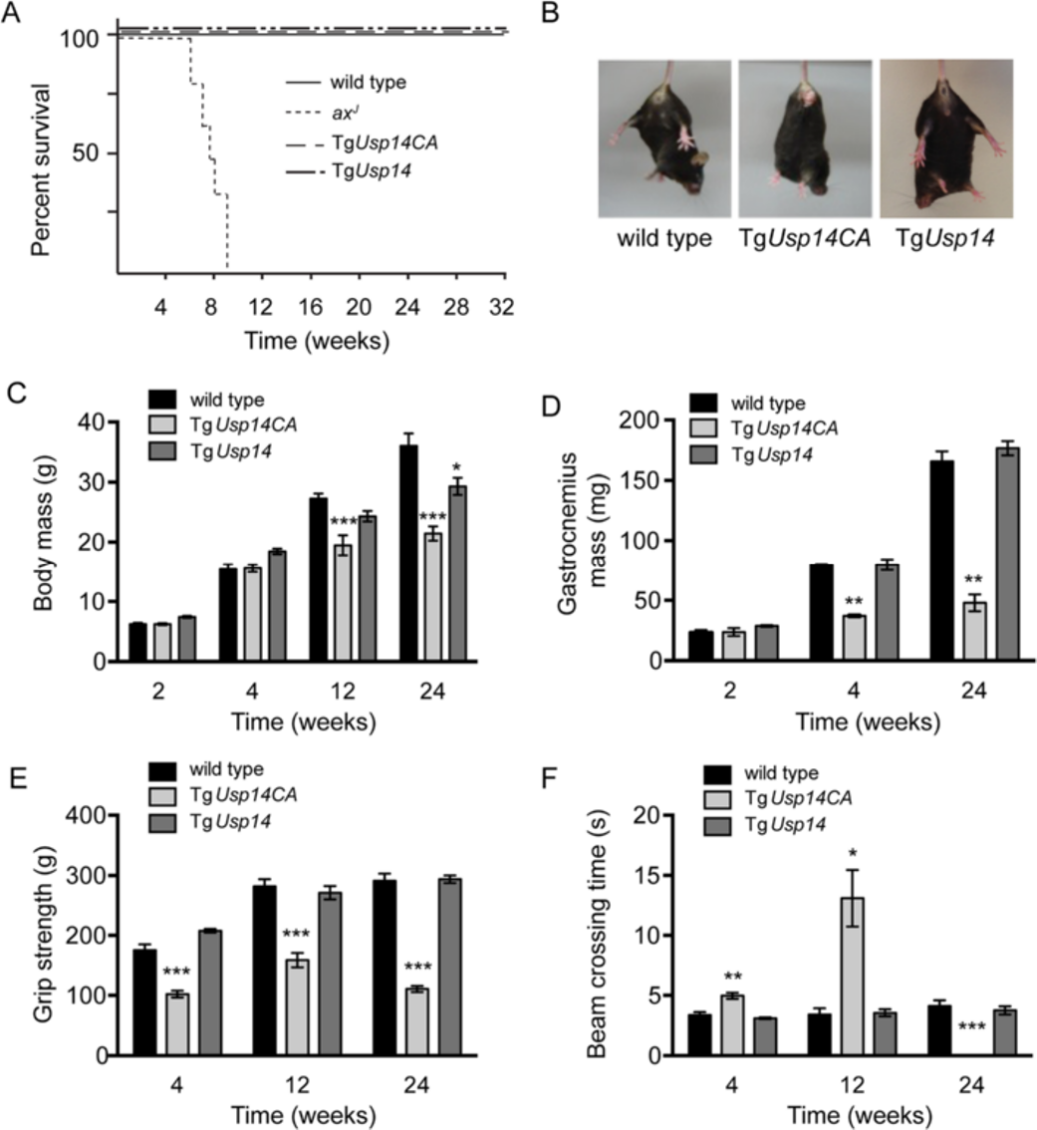
**Neuronal expression of USP14CA leads to decreased body weight, muscle weight, and motor performance. (A)** Survival curves for wild type, Tg*Usp14CA*, Tg*Usp14*, and *ax* ^*J*^ mice. Tg*Usp14* mice express wild type USP14 under the *Thy1.2* promoter and serve as a control for neuronal USP14 overexpression in Tg*Usp14CA* mice. *ax* ^*J*^ mice have a 95% reduction of USP14 in all tissues. **(B)** Tail suspension assays show hind limb clasping in Tg*Usp14CA* mice versus the typical splaying observed in 6-week-old wild type and Tg*Usp14* mice. Assays of **(C)** body mass, **(D)** muscle mass, **(E)** muscle strength and **(F)** motor performance of 2- to 24-week-old wild type, Tg*Usp14CA*, and Tg*Usp14* mice. Muscle strength was assessed by a forelimb grip-force assay and motor performance was assessed by time required to traverse an elevated beam. All data are shown as mean ± SEM. Symbols represent Mann-Whitney tests compared against wild type and corrected for multiple comparisons with a Bonferonni adjustment where appropriate; *p < 0.05, **p < 0.01, ***p < 0.001; n = at least 4 animals per genotype per time point.

Unlike loss of neuronal USP14 in the *ax*^*J*^ mice [21, 22], inhibition of USP14’s DUB activity in the nervous system did not alter the lifespan of the Tg*Usp14CA* mice (Figure 2A). However, Tg*Usp14CA* did have deficits in muscle development, muscle strength, and motor coordination similar to what is observed in *ax*^*J*^ mice, and could be easily distinguished from wild type littermates by 3 weeks of age due to a resting tremor and hind limb clasping upon tail suspension (Figure 2B). The Tg*Usp14CA* mice were significantly smaller than wild type mice by 12 weeks of age (Figure 2C), and had reduced muscle mass (Figure 2D) and fore limb grip strength (Figure 2E) by 4 weeks of age. Similarly, 4-week-old Tg*Usp14CA* mice required more time than wild type controls to cross an elevated beam, and 24-week-old Tg*Usp14CA* mice were completely unable to traverse the beam (Figure 2F). Because the deficits in body mass, muscle development, and motor performance observed in the Tg*Usp14CA* mice were not replicated by overexpression of wild type USP14 in the Tg*Usp14* mice, they were attributed to loss of USP14’s catalytic activity and not to USP14 overexpression.

### USP14’s ubiquitin hydrolase activity supports NMJ structure and function

**Figure 3:**
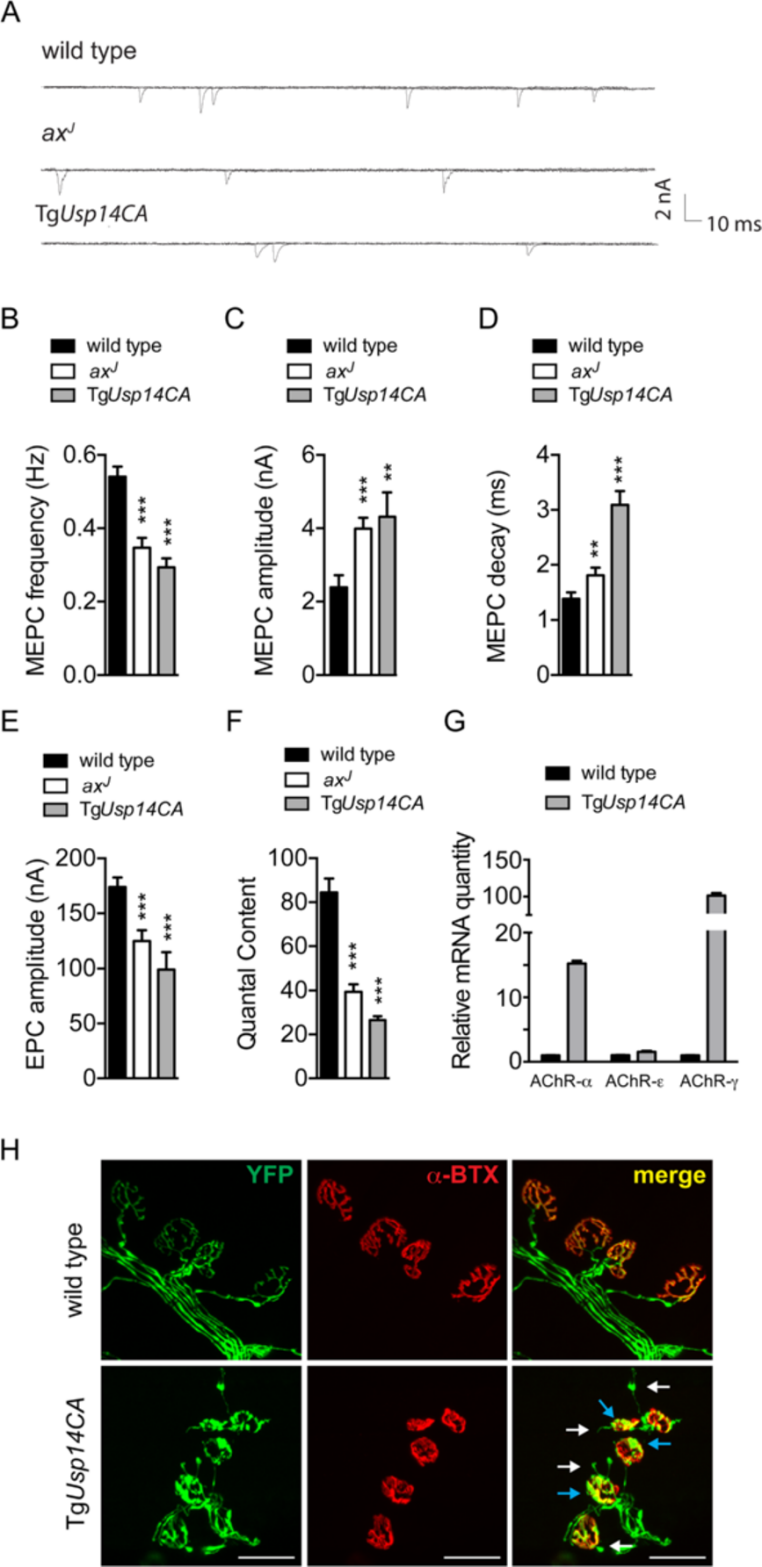
**Expression of Tg** ***Usp14CA*** **causes functional and structural deficits at the NMJ. (A)** Example traces of MEPCs recorded from diaphragms of 4- to 6-week-old wild type, *ax* ^*J*^, and Tg*Usp14CA* mice. **(B)** MEPC frequency in wild type (n = 56 endplates, 10 mice), Tg*Usp14CA* (n = 23 endplates, 4 mice), and *ax* ^*J*^ (n = 75 endplates, 10 mice) muscles. **(C)** MEPC amplitude in wild type (n = 45 endplates, 10 mice), Tg*Usp14CA* (n = 21 endplates, 4 mice) and *ax* ^*J*^ (n = 75 endplates, 10 mice). **(D)** MEPC decay constant in wild type (n = 42 endplates, 10 mice), Tg*Usp14CA* (n = 17 endplates, 4 mice) and *ax* ^*J*^ (n = 23 endplates, 10 mice) muscles. **(E)** Evoked EPC amplitudes in wild type (n = 55 endplates, 10 mice), Tg*Usp14CA* (n = 15 endplates, 4 mice) and *ax* ^*J*^ (n = 75 endplates, 10 mice) muscles. **(F)** Quantal content in wild type (n = 40 endplates, 10 mice), Tg*Usp14CA* (n = 15 endplates, 4 mice) and *ax* ^*J*^ (n = 59 endplates, 10 mice) muscles. **(G)** qPCR of AChR subunits in the gastrocnemius muscles of 4-week-old wild type and Tg*Usp14CA* mice. Data were normalized to wild type and n = at least 3 mice per genotype. All data in (B)-(G) are shown as mean ± SEM. Symbols represent Mann-Whitney tests compared against wild type and corrected for multiple comparisons with a Bonferonni adjustment. *p < 0.05, **p < 0.01, and ***p < 0.001. **(H)** Immunostaining of TA muscles from 4-week-old wild type and Tg*Usp14CA* mice. Axons were visualized via expression of YFP (green) under the neuronal *Thy1.2* promoter and AChRs were labeled with rhodamine-conjugated α-bungarotoxin (α-BTX, red). White arrows indicate ultra-terminal sprouting and blue arrows indicate axonal and terminal swellings, scale bars = 50 μm. See also Additional file 2.

The reduced muscle mass and motor coordination observed in Tg*Usp14CA* mice suggested that neuronal expression of catalytically-inactive USP14 caused deficits in synaptic transmission similar to what we have previously observed in the USP14-deficient *ax*^*J*^ mice [21, 24]. To test this, we used two-electrode voltage clamp to record miniature endplate currents (MEPCs) in diaphragm muscles from 4- to 6-week-old wild type, *ax*^*J*^, and Tg*Usp14CA* mice (Figure 3A). Like the *ax*^*J*^ mice, Tg*Usp14CA* mice had a marked reduction in frequency of MEPCs, and the amplitude and decay constant of MEPCs were increased compared to controls (Figure 3B-D).

A change in MEPC kinetics, such as the increased decay constant observed in Tg*Usp14CA* mice (Figure 3D), often reflects a change in the properties of the postsynaptic receptor. When we examined expression of muscle acetylcholine receptor (AChR) subunits in 4-week-old mice, we found a greater than 100-fold increase in transcripts of the fetal AChR-γ subunit in Tg*Usp14CA* gastrocnemius muscles compared to wild type muscles (Figure 3G). Consistent with the increased decay constant observed at the Tg*Usp14CA* NMJ, AChRs containing the γ-subunit have a longer channel open time than receptors containing the adult ϵ-subunit [26]. The γ-containing receptors and upregulation of the α- and ϵ- AChR subunits observed in the Tg*Usp14CA* mice are also observed following denervation and nerve block [27], and may represent postsynaptic compensation for reduced neurotransmission. However, despite these postsynaptic compensations, the Tg*Usp14CA* endplates displayed reduced synaptic responses upon nerve stimulation (Figure 3E) and decreased quantal content (Figure 3F). We also observed significant pathology when we examined NMJ structure using whole mount immunostaining of the tibialis anterior (TA) muscle. Presynaptic swelling and ultra-terminal sprouting were detectable by 2 weeks of age (see Additional file 2), and by 4 weeks of age, 57% of the Tg*Usp14CA* terminals displayed focal swellings and 56% had ultra-terminal sprouting (Figure 3H).

### Acute inhibition of USP14 leads to reduced neurotransmitter release at the NMJ

**Figure 4:**
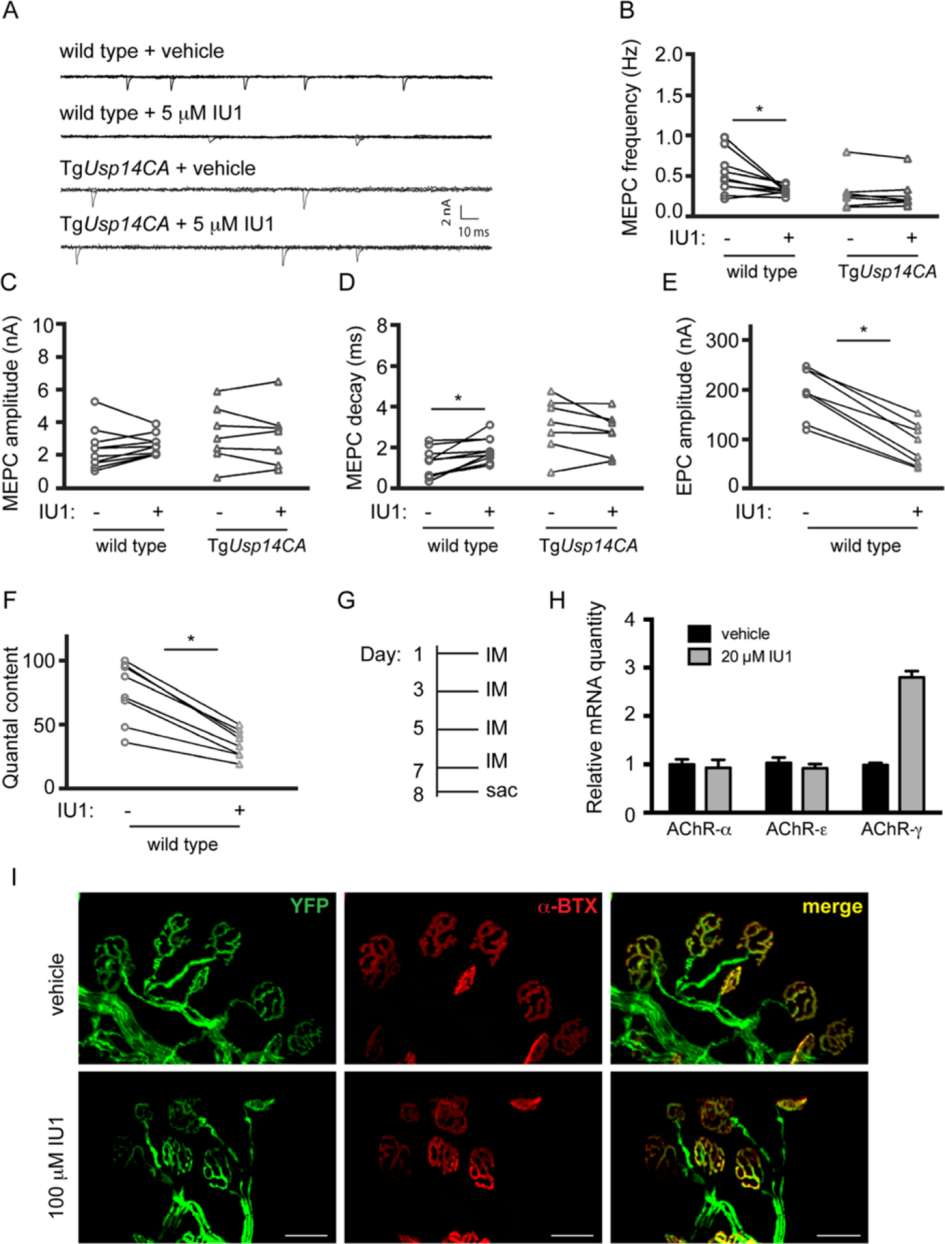
**Acute inhibition of USP14 causes synaptic transmission deficits at the adult NMJ. (A)** Example traces of MEPCs recorded from diaphragm muscle fibers isolated from 4- to 6-week-old wild type (n = 10 endplates, 4 mice) and Tg*Usp14CA* (n = 7 endplates, 3 mice) muscles in the presence of vehicle or the USP14 inhibitor IU1. Quantitation of **(B)** MEPC frequency, **(C)** amplitude, and **(D)** decay constants in muscles from wild type and Tg*Usp14CA* muscles before (-) and after (+) 5 μM IU1 application. Quantitation of **(E)** EPC amplitude and **(F)** quantal content in wild type muscles (n = 10 endplates, 4 mice) before (-) and after (+) addition of 5 μM IU1. In (B)-(F), symbols represent Wilcoxon matched-pairs signed rank tests, where *p < 0.05 **(G)** Schematic of experimental design, where adult wild type mice (12- to 14-weeks-old) were given intramuscular injections of 100 μL of 100 μM IU1 or vehicle every other day for 1 week, and **(H)** the abundance of AChR subunit mRNA in gastrocnemius muscles was compared using qPCR. Data are shown as mean ± SEM and were normalized to vehicle treatment. n = 3 muscles per condition, run in triplicate. **(I)** Whole mount immunostaining of gastrocnemius muscles from wild type mice given intramuscular injections of IU1 or vehicle as described above. Endplates were labeled with rhodamine-conjugated α-BTX, and motor neurons were visualized via expression of YFP under the *Thy1.2* promoter. Scale bars = 50 μm.

To differentiate between abnormal development and an ongoing need for USP14’s DUB activity in synaptic transmission at the adult NMJ, we measured spontaneous and evoked transmission following acute treatment with the USP14 inhibitor IU1 in phrenic nerve/diaphragm preparations from adult wild type and Tg*Usp14CA* mice. Example traces for each condition are shown in Figure 4A. IU1 treatment of wild type NMJs replicated the decrease in MEPC frequency (Figure 4B) and the increase in the MEPC decay constant (Figure 4D) observed in the *ax*^*J*^ and Tg*Usp14CA* mice (Figure 3B and D). In contrast, we did not observe any effect of IU1 treatment on Tg*Usp14CA* NMJs, indicating that the IU1-mediated alterations in synaptic transmission were due to inhibition of USP14’s DUB activity. Acute inhibition of USP14 in wild type mice also recapitulated the deficits in EPC amplitude and quantal content (Figure 4E and F) observed in Tg*Usp14CA* mice (Figure 3E and F). Only the increase in MEPC amplitude observed in *ax*^*J*^ and Tg*Usp14CA* mice was not mimicked by IU1 treatment (Figure 4C). These data indicate that the synaptic deficits observed in Tg*Usp14CA* mice are not due to aberrant development, but rather reflect an ongoing need for USP14’s DUB activity at the adult NMJ.

The increase in the MEPC decay constant in Tg*Usp14CA* mice was associated with increased expression of the fetal AChR-γ subunit in gastrocnemius muscles (Figure 3G). Similarly, 1 week of IU1 injections into the gastrocnemius muscles of adult, wild type mice (Figure 4G) caused an increase in AChR-γ abundance compared to what was observed in vehicle-injected control muscles (Figure 4H), indicating that USP14-inhibition at the adult NMJ is sufficient to cause induction of the embryonic AChR-γ subunit. We also examined NMJ structure following IU1 treatment. Unlike in Tg*Usp14CA* mice (Figure 3H), no swollen terminals or ultra-terminal sprouts were observed in IU1-treated wild type mice, and the endplates retained their mature, arborized appearance (Figure 4I). These experiments demonstrate that abnormal terminal structure is not a prerequisite for deficient synaptic transmission, and suggests that these deficits may have distinct underlying mechanisms.

### Inhibition of USP14’s DUB activity causes accumulation of K63-linked ubiquitin chains

The role of USP14 in the maintenance of ubiquitin homeostasis is well established, so we reasoned that aberrant ubiquitin signaling might underlie the neuromuscular phenotype of the Tg*Usp14CA* mice and the synaptic deficits caused by acute inhibition of USP14. Consistent with USP14’s ubiquitin hydrolase activity, we found an increase in ubiquitinated proteins in the spinal cords of Tg*Usp14CA* mice compared to controls and in cortical neurons treated with IU1 (see Additional file 3).

**Figure 5:**
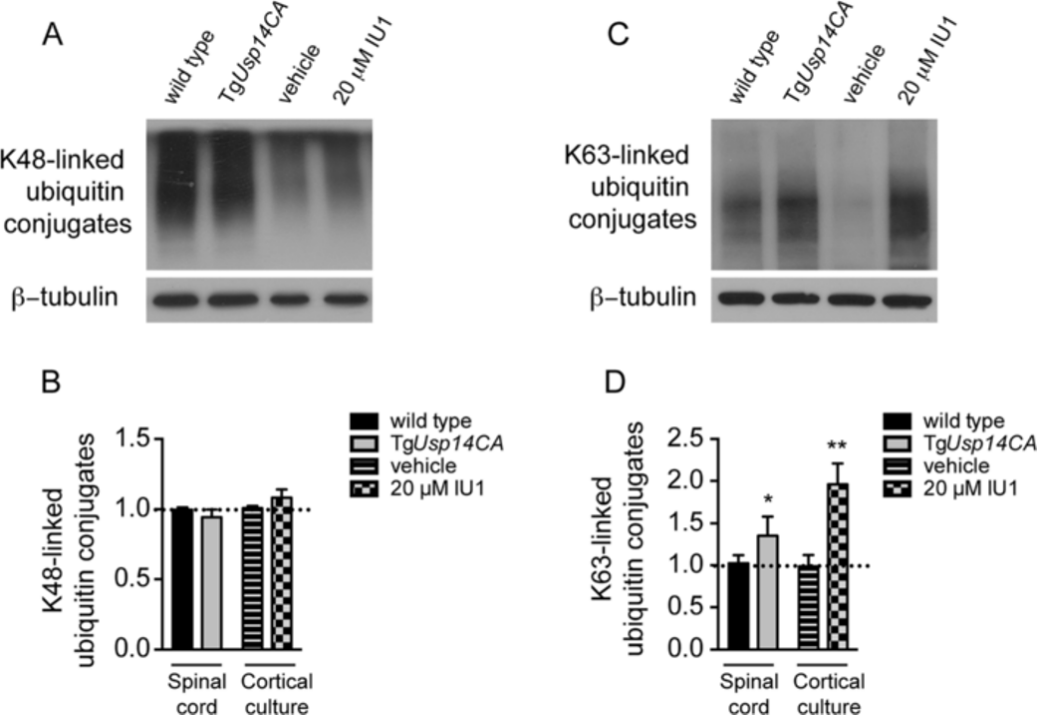
**Loss of USP14’s ubiquitin hydrolase activity results in accumulation of non-proteasomal associated ubiquitin conjugates. (A)** Representative immunoblot of spinal cord extracts from 4- to 6-week-old wild type and Tg*Usp14CA* mice and cortical neurons treated with vehicle or 20 μM IU1 for 24 h and probed with an antibody specific for K48-linked ubiquitin chains. **(B)** Quantitation of (A). **(C)** As in (A) except with anti-K63-linked ubiquitin chain antibody. β-tubulin was used as a loading control in all experiments. **(D)** Quantitation of (D). Data are shown as mean ± SEM, and n = at least 3 animals per genotype or condition run in duplicate. Symbols represent Mann-Whitney tests, where *p < 0.05 and **p < 0.01. See also Additional file 3.

Although we found no difference in the catalytic capacity of Tg*Usp14CA* and wild type proteasomes towards the fluorogenic Boc-LRR-AMC substrate (Figure 1G), this increase in ubiquitin conjugates was consistent with decreased turnover of ubiquitinated proteins by the proteasome. Because the Boc-LRR-AMC substrate is not ubiquitinated and can bypass the proteasome’s regulatory particle and enter the catalytic core directly, the turnover of this substrate may not accurately reflect the turnover of ubiquitinated proteins *in vivo*. However, when we measured the abundance of proteasomal-targeting K48-linked ubiquitin conjugates, which serve as a robust and sensitive marker of ubiquitin proteasome system function [28], we found no difference between wild type and Tg*Usp14CA* spinal cords or between IU1- and vehicle-treated neurons (Figure 5A and B). Instead, we observed increased K63-linked ubiquitin conjugates in both Tg*Usp14CA* spinal cords and IU1-treated neurons (Figure 5C and D). K63-linked ubiquitin chains do not appear to target proteins for proteasomal degradation [29–31], but, instead, regulate receptor internalization, endosomal sorting, and intracellular signaling [29].

### USP14’s DUB activity regulates the MLK3 signaling cascade

**Figure 6:**
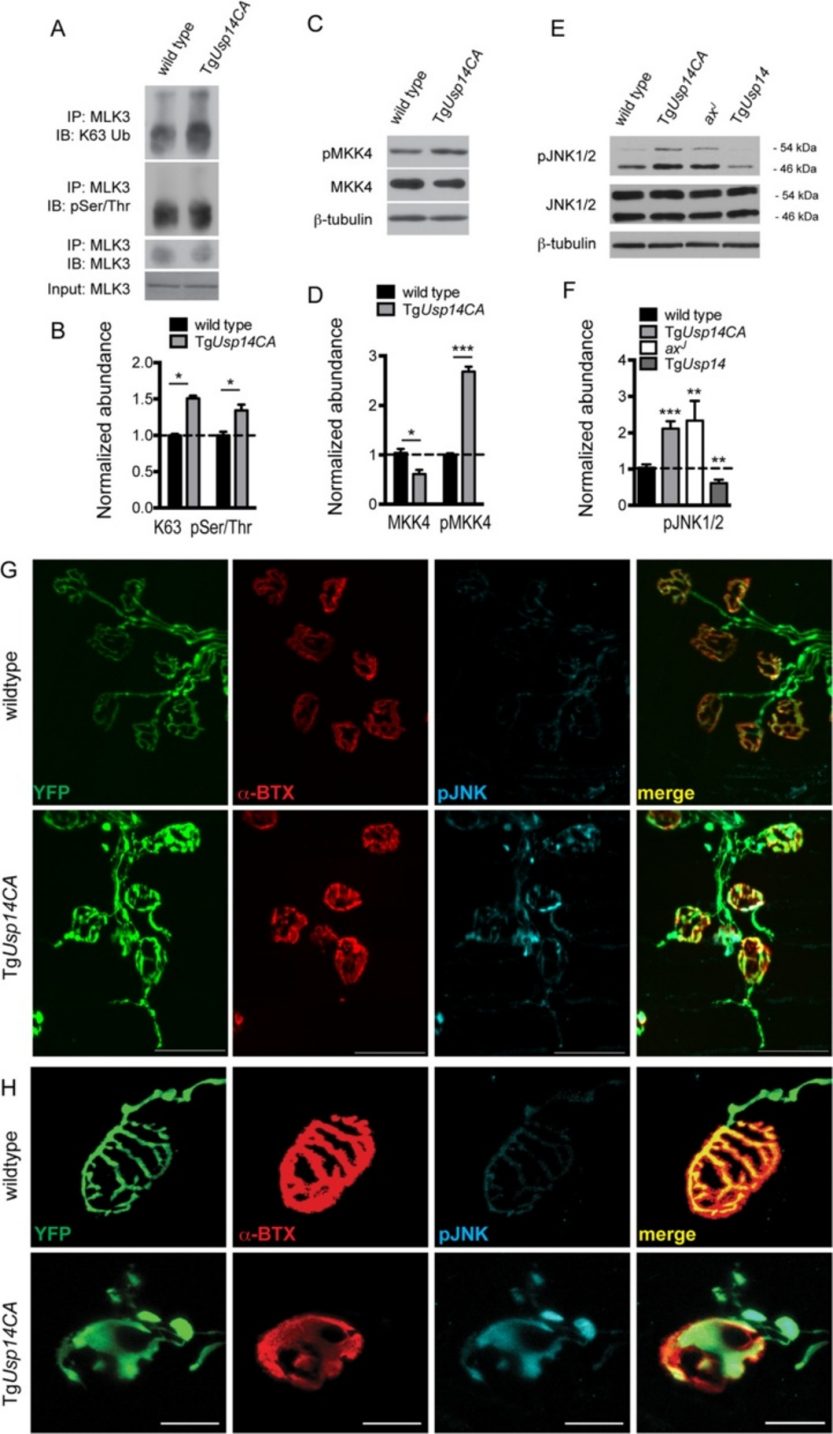
**Inhibition of USP14’s DUB activity leads to enhanced activation of the MLK3 signaling cascade. (A)** Representative immunoprecipitates (IP) of MLK3 from spinal cord lysates from 4- to 6-week-old wild type and Tg*Usp14CA* mice, immunoblotted (IB) for K63-linked ubiquitin, phospho-serine/threonine, and MLK3. **(B)** Quantitation of (A), K63- and pSer/Thr- modified MLK3 was normalized to total immunoprecipitated MLK3. **(C)** Representative immunoblots of wild type and Tg*Usp14CA* spinal cord extracts probed for pMKK4 and total MKK4. β-tubulin was used as a loading control. **(D)** Quantitation of (C), pMKK4 was normalized to MKK4. **(E)** Representative immunoblots from spinal cords from 4- to 6-week-old wild type, Tg*Usp14CA*, *ax* ^*J*^, and Tg*Usp14* mice, probed for pJNK1/2 and total JNK1/2. JNK 1 and 2 migrate to 46 and 54 kDa, respectively. β-tubulin was used as a loading control. **(F)** Quantitation of (E), pJNK was normalized to JNK and quantitation includes both the 46 and 54 kDa bands. Data in (B), (D), and (F) are shown as mean ± SEM, and n = 3 animals per genotype. Symbols represent Mann-Whitney tests compared against wild type mice and corrected for multiple comparisons with a Bonferonni adjustment where appropriate; *p < 0.05, **p < 0.01, ***p < 0.001. **(G)** Representative images of whole-mount immunostaining of TA muscles from 4-week-old wild type and Tg*Usp14CA* mice using a pJNK antibody (blue). AChRs were labeled with rhodamine-conjugated α-BTX (red), and motor neuron axons were visualized via expression of YFP under the *Thy1.2* promoter (green). Scale bars = 50 μm. **(H)** As in (G), except that scale bars = 20 μm. See also Additional file 4.

K63-linked ubiquitination of MLK3 leads to dimerization and auto-phosphorylation on threonine 277/serine 281 [6]. This auto-phosphorylation enables MLK3 to phosphorylate mitogen-activated protein kinase kinase 4 (MKK4), which, in turn, phosphorylates JNK. Because MLK3 is activated by K63-linked ubiquitination and elevated pJNK has been demonstrated to cause terminal swelling [32] and sprouting [33] at the NMJ that is similar to what we observed in the Tg*Usp14CA* mice (Figure 3H), we investigated whether this pathway was hyperactivated in the Tg*Usp14CA* mice. When we immunoprecipitated MLK3 from spinal cord extracts, we observed increased K63-linked ubiquitination and enhanced serine/threonine phosphorylation of high molecular weight MLK3 species in Tg*Usp14CA* lysates compared to wild type lysates (Figure 6A and B). We also observed increased phosphorylation of MLK3’s target MKK4, even though total MKK4 abundance was decreased in Tg*Usp14CA* mice relative to controls (Figure 6C and D). Although we observed a significant decrease in *Mkk4* mRNA in Tg*Usp14CA* spinal cords compared to wild type spinal cords (see Additional file 4), the magnitude of the effect may not explain the 39% reduction in MKK4 protein.

When compared to wild type mice, we observed increased pJNK1/2 in spinal cord extracts of Tg*Usp14CA* and *ax*^*J*^ mice and decreased pJNK1/2 in spinal cord extracts of Tg*Usp14* mice, (Figure 6E and F) indicating that USP14’s DUB activity can bidirectionally modulate JNK activation, likely by controlling MLK3 ubiquitination. Notably, there was no change in the activation of the MAP kinases p38 and ERK in the spinal cords of Tg*Usp14CA* mice compared to wild type mice, and, although pJNK can induce cell death, there was no evidence of apoptosis in spinal cords of Tg*Usp14CA* mice (see Additional file 4). We next performed whole mount immunostaining of TA muscles of wild type and Tg*Usp14CA* mice using an antibody against pJNK (Figure 6G and H) and found a significant correlation between pJNK staining and Tg*Usp14CA* terminal pathology. In the Tg*Usp14CA* mice, 94% of the terminal swellings and 90% of the ultra-terminal sprouts were pJNK positive.

### Elevated pJNK contributes to the structural and functional deficits caused by loss of USP14’s DUB activity

**Figure 7:**
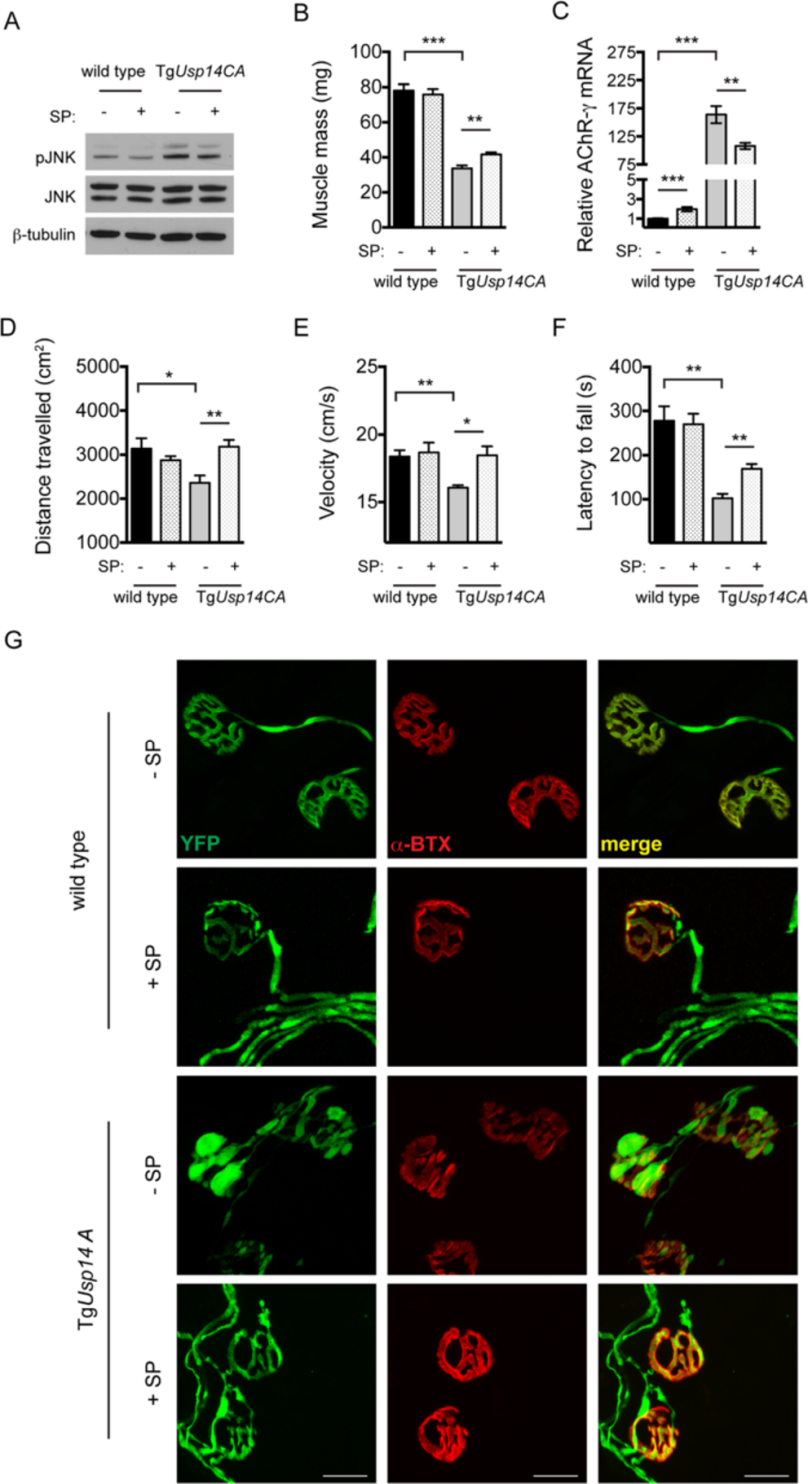
**Inhibition of pJNK improves the structural and functional deficits caused by inhibition of USP14’s DUB activity. (A)** Representative immunoblots of pJNK1/2 and JNK1/2 in spinal cords from 5-week-old wild type or Tg*Usp14CA* mice following 2 weeks of IP injections of the JNK inhibitor SP600125 (SP, +) or vehicle control (DMSO, -). β-tubulin was used as a loading control. Wild type mice given SP had pJNK levels that were 71.2 ± 10.3% (p < 0.01, Mann-Whitney test) of what was observed in vehicle-treated wild type controls and SP-treated Tg*Usp14CA* mice had pJNK levels that were 66.6 ± 19.4% (p < 0.01, Mann-Whitney test) of vehicle-treated Tg*Usp14CA* mice. **(B)** Gastrocnemius muscle masses from male wild type or Tg*Usp14CA* mice treated with vehicle alone (-) or SP (+) as described above. The same effect was observed in female mice. **(C)** Relative *AChR-γ* mRNA abundance in gastrocnemius muscles. **(D)** Total distance travelled and **(E)** ambulatory velocity during 10 min in an open field. **(F)** Latency to fall from beam in the rotarod assay. For (B)-(F), all data are shown as mean ± SEM and n = 5 to 7 animals per condition. Symbols represent Mann-Whitney tests corrected for multiple comparisons with a Bonferonni adjustment, where *p < 0.05, **p < 0.01, and ***p < 0.001. **(G)** High magnification images of whole-mount immunostaining of TA muscles from mice described above. AChRs were labeled with rhodamine-conjugated α-BTX (red), and motor neuron axons were visualized via expression of YFP under the *Thy1.2* promoter (green). Scale bars = 20 μm.

Because excess pJNK at the NMJ has previously been shown to cause motor endplate disease [33] and JNK inhibition causes increased neurotransmitter release in the central nervous system [34], we hypothesized that the elevated pJNK observed in the Tg*Usp14CA* spinal cords and motor neuron terminals may have caused deficits in NMJ structure and function. To test this, we administered the JNK inhibitor SP600125 (SP) to 3-week-old wild type and Tg*Usp14CA* mice via intraperitoneal (IP) injections. Daily IP injections were given at a dose of 16 mg/kg for 2 weeks, and control mice received an equivalent volume of DMSO vehicle alone. SP inhibits JNK by reversibly occupying its ATP binding pocket to prevent kinase activity towards its substrates. Because JNK is autophosphorylated, SP causes a reduction of pJNK (Figure 7A) that is equivalent to the reduction of activated JNK substrates [35]. On average, we observed an approximately 30% reduction of pJNK in the spinal cords of SP-treated mice compared to their vehicle-treated counterparts (wild type: normalized pJNK abundance 1.01 ± 0.07 for vehicle-treated mice versus 0.71 ± 0.10 for SP-treated mice, p < 0.01, Mann-Whitney test; Tg*Usp14CA*: 1.03 ± 0.08 for vehicle-treated mice versus 0.67 ± 0.19 for SP-treated mice, p < 0.01, Mann-Whitney test).

Although we found no difference between the gastrocnemius muscle masses of wild type mice treated with SP or vehicle alone, SP injections resulted in a significant increase in the gastrocnemius mass of Tg*Usp14CA* mice (Figure 7B). This effect was observed in both male and female Tg*Usp14CA* mice but was not significant when male and female data were pooled because of the disparity in muscle hypertrophy between genders. We also observed a significant decrease in the abundance of *AChR-γ* mRNA in SP-treated Tg*Usp14CA* mice compared to vehicle-treated Tg*Usp14CA* mice (Figure 7C). Although there was a significant upregulation of *AChR-γ* mRNA in SP-treated wild type mice compared to vehicle-treated controls, this effect cannot explain the decrease in AChR-γ transcript abundance observed in Tg*Usp14CA* mice. Because neuronal activity can promote muscle hypertrophy and AChR transcript abundance is inversely proportional to synaptic transmission, both the increase in muscle mass and the decrease in AChR transcript abundance observed in Tg*Usp14CA* mice following JNK inhibition are consistent with improved presynaptic NMJ function.

When we compared locomotion while exploring a novel, open field, we found a significant decrease in distance traveled (Figure 7D) and ambulatory velocity (Figure 7E) in Tg*Usp14CA* mice compared to wild type mice. However, SP-treated Tg*Usp14CA* mice traveled as far and as fast as wild type mice in the open field (Figure 7D and E). In the more demanding rotarod assay, the performance of the SP-treated Tg*Usp14CA* mice was improved over vehicle-treated Tg*Usp14CA* mice, but was still significantly worse than wild type mice (Figure 7F). We next used whole mount immunostaining to determine if the improvements in NMJ function observed in the Tg*Usp14CA* mice following SP treatment were accompanied by improved NMJ structure. We found that, while the NMJs of SP-treated Tg*Usp14CA* mice were still abnormal compared to wild type mice, there was an appreciable decrease in presynaptic swelling and an increase in endplate arborization compared to vehicle-treated Tg*Usp14CA* mice (Figure 7G).

## Discussion

The purpose of this study was to determine whether USP14’s catalytic activity is required for NMJ function and to investigate the effects of USP14 inhibition on proteasomal-dependent and –independent ubiquitin signaling in the nervous system. Indeed, our results demonstrate that USP14’s ubiquitin hydrolase activity modulates NMJ development and synaptic transmission (Figures 3 and 4) and regulates proteasomal-independent ubiquitin signaling (Figures 5 and 6). We observed enhanced K63-linked ubiquitination of MLK3 and hyperactivation of its downstream kinase JNK in the spinal cords of mice expressing catalytically-inactive USP14 (Figure 6), and JNK inhibition significantly improved both NMJ structure and motor function in these mice (Figure 7), demonstrating that the regulation of pJNK by USP14 is critical to nervous system structure and function. In addition, we uncovered an ongoing need for USP14’s DUB activity at adult NMJs by demonstrating that the synaptic transmission deficits in mice expressing USP14CA (Figure 5) were replicated by acute inhibition of USP14 (Figure 4).

### USP14 regulates proteasome-independent ubiquitin signaling

The increases in ubiquitin conjugates observed in the spinal cords of Tg*Usp14CA* mice and in neuronal cultures when USP14 is pharmacologically inhibited (see Additional file 3) mimic the effects on ubiquitin conjugates when the proteasome is inhibited [29, 30]. However, the level of K48-linked ubiquitin conjugates was unchanged in spinal cords from Tg*Usp14CA* mice compared to wild type mice and in cortical neurons with and without IU1 (Figure 5A and B). Because K48-linked ubiquitin chains are a robust biomarker of ubiquitin proteasome system function [28], and we found no difference in the catalytic capacity of Tg*Usp14CA* and wild type proteasomes *in vitro* (Figure 1G), it is unlikely that USP14 inhibition in the nervous system causes global alterations in proteasome activity. Instead, we observed an increase in K63-linked ubiquitin conjugates, which do not accumulate following proteasome inhibition [29, 30], in both Tg*Usp14CA* spinal cords and IU1-treated cortical neurons (Figure 5C and D), suggesting that USP14 disassembles non-proteasomal-targeting ubiquitin chains.

Expression of catalytically inactive USP14 in the nervous systems of Tg*Usp14CA* mice led to enhanced K63-linked ubiquitination of MLK3 (Figure 6A and B), which has previously been demonstrated to induce its dimerization, auto-phosphorylation, and ability to phosphorylate MKK4 [6]. Consistent with this finding, we observed enhanced phosphorylation of MLK3 (Figure 6A and B) and MKK4 (Figure 6C and D), as well as MKK4’s target JNK (Figure 6E and F), in Tg*Usp14CA* spinal cords. We also observed prominent pJNK immunoreactivity in the swollen terminals and within the ultra-terminal sprouts of Tg*Usp14CA* motor neurons (Figure 6G). In fact, treatment with the JNK inhibitor SP600125 improved the muscle development (Figure 7B), motor function (Figure 7D-F), and NMJ structure (Figure 7G) of the Tg*Usp14CA* mice, even though we began administration of the drug after the neuromuscular phenotype was established (Additional file 2). More robust JNK inhibition or beginning JNK inhibitor treatment at an earlier age may have led to a more complete rescue of the Tg*Usp14CA* phenotype. Alternatively, the JNK pathway may be one of multiple pathways that are regulated by USP14’s catalytic activity. Support for the later explanation comes from studies of the *Drosophila highwire (hiw)* mutant, which has NMJ pathology and synaptic transmission deficits that are similar to what we observed in Tg*Usp14CA* mice [33]. Genetic inactivation of the JNK signaling cascade in the *hiw* mutants corrects their NMJ pathology, but their deficits in synaptic transmission persist.

### USP14 has an ongoing role in synaptic transmission

We observed decreased MEPC frequency, EPC amplitude, and quantal content at the NMJs of Tg*Usp14CA* and *ax*^*J*^ mice (Figure 3A-F). Surprisingly, these deficits were mimicked by acute inhibition of USP14 at the NMJs of adult, wild type mice (Figure 4A-F), likely indicating that USP14 has an ongoing role in synaptic transmission and that the synaptic deficits in Tg*Usp14CA* and *ax*^*J*^ mice do not arise from aberrant development. This conclusion is supported by the finding that intramuscular injections of IU1 were sufficient to cause alterations in AChR mRNA expression but did not alter NMJ structure (Figure 4G-I), suggesting that the structural and functional deficits at the Tg*Usp14CA* NMJs arise through different mechanisms. Because synaptic proteins are turned over very slowly *in vivo*, with an average turnover rate of 9 days [36], and we found no evidence of proteasome dysfunction in the Tg*Usp14CA* mice or in IU1-treated neurons, the rapid effects of IU1 on synaptic transmission are unlikely to result from altered protein turnover. Instead, the rapid effect of USP14 inhibition on MEPC frequency, the accumulation of K63-linked chains upon USP14 inhibition (Figure 6), and the regulation of JNK phosphorylation by USP14 activity (Figure 7E and F) all support a model in which USP14 dynamically deubiquitinates proteins to affect their activity and not their abundance.

## Conclusion

In this study, we demonstrated that USP14’s catalytic activity is required for NMJ structure and function, and provided evidence that USP14 regulates non-proteasomal ubiquitin signaling in the nervous system. Although our results are consistent with a recently published report demonstrating that USP14 removes K63-linked ubiquitin chains from Dvl2 [15], both studies raise the question of how a proteasome-associated DUB could disassemble proteasome-independent ubiquitin chains. Further study will be required to determine if proteasome-bound USP14 can interact with K63-linked ubiquitin chains or, alternatively, whether USP14’s DUB activity can be stimulated by a novel binding partner, allowing it to be active when not bound to the proteasome. In either case, our findings demonstrate a novel role for USP14 and establish non-canonical ubiquitin signaling as an important contributor to both the long-term health of the nervous system and synaptic transmission.

## Methods

### Animals

C57BL/6J mice (wild type), *Usp14*^*axJ*^ mice (Jackson Laboratories, Bar Harbor, MA) Thy1*-YFP* mice (16JRS, Jackson Laboratories), transgenic mice expressing wild type USP14 (Tg*Usp14*) [22], and transgenic mice expressing catalytically inactive USP14 (Tg*Usp14CA*) have been maintained in our breeding colony at the University of Alabama at Birmingham, which is fully accredited by the Association for Assessment and Accreditation of Laboratory Animal Care International. All mouse lines were maintained on a C57BL/6J background. Homozygous *Usp14*^*axJ*^ mice (which we refer to as *ax*^*J*^ mice) were generated by intercrossing heterozygous *ax*^*J*^ siblings. Research was conducted without bias toward the sex of animals used for each study, and male and female mice were used equally. All research complied with the United States Animal Welfare Act and other federal statutes and regulations relating to animals and experiments involving animals and adhered to principles stated in the Guide for the Care and Use of Laboratory Animals, United States National Research Council.

### Construction of TgUsp14CA transgene

The full-length *Usp14* cDNA, including the *Usp14* Kozak consensus sequence, was generated by using reverse transcription-PCR (RT-PCR). USP14’s active site cysteine (C114) was replaced with an alanine residue via PCR site-directed mutagenesis. The cDNA was cloned into the *XhoI* site of the *pThy1.2* expression cassette (gift from Dr. Pico Caroni at the Friedrich Institute, Basel, Switzerland). The transgene was excised from the vector by using *EcoRI* and *NdeI* and prepared for microinjection via standard procedures.

### RNA transcriptome analysis

Total RNA was isolated from hippocampal lysates of 4-week-old wild type and Tg*Usp14CA* mice using RNA-STAT60 (Tel-Test, Friendswood, TX). Poly(A) RNAs were subsequently purified using an RNeasy Mini Kit (Qiagen, Valencia, CA). cDNAs were generated and paired-end sequencing was performed at Hudson Alpha (Huntsville, AL).

### Neuronal culture

Dissociated cortical cultures were prepared as described [37]. Cultured neurons were treated with the USP14 inhibitor IU1 (20 μM) or an equivalent volume of vehicle (DMSO) at 10 to 11 days *in vitro*, and protein was harvested 24 h later as described below.

### Isolation of proteins

Mice 4- to 6- weeks of age were deeply anesthetized via isoflurane prior to rapid decapitation. Tissues were removed and homogenized in 1 to 3 mL of buffer containing 50 mM Tris, 150 mM NaCl, 5 mM MgCl_2,_ 2 mM N-ethylmaleimide, 0.5% SDS, Complete protease inhibitors (Roche, Indianapolis, IN) and phosphatase inhibitor cocktail I (Sigma Aldrich, St. Louis, MO), pH 7.5. PR-619 (Life Sensors, Malvern, PA) was added to a final concentration of 50 μM to inhibit a wide range of DUBs. After homogenization, tissues were centrifuged at 17,000 × g for 10 min at 4°C, and supernatants were removed and immediately frozen at –80°C. Protein concentrations were determined by using the bicinchoninic acid (BCA) protein assay kit from Pierce (Rockford, IL).

### Isolation of proteasomes and assays of proteasome activity

Proteasomes were isolated and assayed as described [9].

### Immunoblotting

Protein electrophoresis and blotting was performed as described [24]. Immunoblots probed for ubiquitin were pretreated with 0.1% gluteraldehyde for 20 min prior to blocking.

### Immunoprecipitation

Proteins were isolated as described above in a modified RIPA buffer containing 0.5% SDS and heated to 95°C for 5 min. Protein lysates were diluted 1:10 in a buffer containing 25 mM Tris and 150 mM NaCl with a pH of 7.2 and incubated with 10 μg of MLK3 antibody overnight at 4°C with constant agitation. The antibody/antigen complex was added to 100 μL of Immobilized Protein A/G resin slurry (Thermo Scientific, Waltham, MA) and incubated with constant agitation at 4°C for 6 hours. Immunoprecipitates were collected by centrifugation according to manufacturer instructions.

### Antibodies

The following antibodies were used: USP14 [9]; β-tubulin (Developmental Studies Hybridoma Bank, Iowa City, IA); Rpt1 and MLK3 (Santa Cruz Biotechnologies, Dallas, TX); Ubiquitin (UAB Hybridoma Facility, Birmingham, AL), K48 Ubiquitin and K63 Ubiquitin (Millipore, Billerica, MA); pMKK4, MKK4, pJNK, and JNK (Cell Signaling Technology, Danvers, Massachusetts).

### Quantitation of immunoblots

Blots were quantitated using ImageJ software (NIH, Bethesda, MD). Each value represents the average and standard error from at least two blots using at least three different animals of each genotype.

### Labeling of proteasome-associated DUBs

Activity of proteasomal DUBs was assayed as previously described [22].

### Immunocytochemistry (ICC)

ICC of PFA-fixed brain sections was performed as described previously [38]. Briefly, following perfusion with Tyrode’s solution and 4% PFA, serial coronal sections of brain (50 μm) were prepared, and free-floating immunohistochemistry was performed. Spinal cords were paraffin embedded. USP14 was detected using Alexa Fluor anti-rabbit 594 antibody (Invitrogen, Carlsbad, CA). Sections were washed in PBST containing DAPI (Invitrogen) to detect nuclei. Free-floating sections were mounted on gelatinized slides and dehydrated in graded ethanol and xylenes. Coverslips for all sections were secured in Prolong-gold anti-fade mounting media (Invitrogen). TUNEL staining was performed using the Roche TUNEL labeling kit per manufacturer instructions (Roche Diagnostics Corporation, Indianapolis, IN).

### Grip strength test

Forelimb grip strength was determined using an animal grip strength system (SDI, San Diego, CA). Each trial consisted of five repetitions of this assay (n > 5 mice per genotype per time point).

### Elevated beam assay

Ability to traverse an elevated beam 2 cm in diameter was assessed as previously described [22], n > 5 mice per genotype per time point.

### Rotarod assay

Motor coordination was tested by placing mice on a rotating rod (ENV-575, Med Associates, St. Albans, VT), which accelerated from 3.5 rpm to 35 rpm over a 5-min period. Latency to fall was recorded over 3 trials, each separated by 1 h, and the individual trials for each animal were averaged.

### Open field assay

Animals were handled 1 day prior to open field testing. Locomotor activity was measured in an open field chamber (43.2 cm × 43.2 cm × 30.5 cm) for 15 min by an automated video tracking system. The first 5 min were not analyzed to account for habituation to the chamber.

### NMJ immunostaining and confocal imaging

Whole mount immunostaining of the tibialis anterior (TA) muscle was performed as described [23], with minor modifications. The TA muscle was immersed in ice-cold PBS containing 2% PFA for 1 h following dissection and immediately teased into thin bundles. Muscle bundles were then transferred to PBS containing 1% PFA and 1% Triton and permeabilized overnight at 4°C with constant rocking. To improve visualization of axons and ultra-terminal sprouting, all mice used for NMJ immunostaining carried the *Thy1-YFP* transgene in addition to the transgene of interest. For pJNK immunostaining, muscle bundles were incubated with primary antibody (pJNK, #81E11, Cell Signaling Technology, Danvers, MA) for 5 days at 4°C with constant rocking. Images were captured using a Zeiss LSM 510 Meta confocal microscope (Carl Zeiss, Oberkochen, Germany).

### Quantitative PCR

RNA isolation and quantitative PCR (qPCR) were performed as described [23]. Individual gene assay kits were purchased from Applied Biosystems for each of the RNAs analyzed. n = at least 3 animals per genotype per time point run in triplicate.

### Electrophysiology

Two-electrode voltage clamp was performed as described [24]. Diaphragms, with ribs and intact phrenic nerves, were dissected from 5- to 6-week-old mice. All experiments were performed at room temperature. For experiments using IU1, measurements were taken in the presence of vehicle alone prior to the introduction of 20 μM IU1 into the perfusing solution.

### Intramuscular injections of IU1

Adult mice (12- to 14-weeks old) were lightly anesthetized with isoflurane for injections into the gastrocnemius muscle. Injections (100 μL) contained IU1 (100 μM) or vehicle with 0.1% India blue ink, to ensure correct injection location, and 0.05 mg/kg RIMADYL (Pfizer, New York City, NY), to reduce discomfort, in sterile PBS. Each animal (n = 3) received vehicle injection and IU1 injection into the left and right gastrocnemius muscles, respectively.

### Intraperitoneal injections of SP600125

3-week-old wild type and Tg*Usp14CA* mice were given daily intraperitoneal injections of the JNK inhibitor SP600125 (Fisher Scientific, Rockford, IL) for two weeks. SP600125 was dissolved in DMSO at a concentration of 91 mM and this solution was directly administered at a dose of 16 mg/kg using a 25 μL Hamilton syringe (Hamilton Company, Reno, NV). This procedure was used to minimize the injection volume, as SP600125 is not soluble in aqueous solutions and DMSO was not tolerated at higher doses. Animals were weighed every other day to adjust doses and to monitor potential adverse effects of the injections. At the doses given, both DMSO and SP600125 were well tolerated.

## Electronic supplementary material

Additional file 1: Figure 1: USP14CA is expressed specifically in the nervous system. Description of Data: (A) Developmental time course of USP14 expression in spinal cords of wild type (-) and Tg*Usp14CA* (+) mice on postnatal days (P) 1-180 showing robust expression of the transgene by P8. β-tubulin was included as a loading control. (B) Representative immunoblots of USP14 from 4- to 6-week old wild type (-) and Tg*Usp14CA* (+) mice demonstrating neuronal expression of the transgene. USP14 overexpression is assumed to reflect transgene expression. β-tubulin was included as a loading control (C) Representative immunostaining for USP14 (red) and DAPI (blue) in cerebral cortices and spinal cords of 8-week-old wild type and Tg*Usp14CA* mice. Scale bar = 100 μm. (PDF 13 MB)

Additional file 2: Figure3: Tg*Usp14CA* mice have abnormal NMJ structure starting at 2 weeks of age. Description of data: (A) Whole-mount immunostaining of TA muscles from wild type and Tg*Usp14CA* mice in 1- and 2-week-old. Motor neuron axons were stained with antibodies against neurofilament and synaptophysin (green), and AChRs were labeled with rhodamine-conjugated α-bungarotoxin (α-BTX, red). White arrows indicate ultra-terminal sprouting and blue arrows indicate axonal swellings, scale bars = 50 μm. (PDF 3 MB)

Additional file 3: Figure 5: Genetic inactivation and pharmacological inhibition of USP14’s ubiquitin hydrolase activity lead to increased ubiquitin conjugates. Description of data: (A) Representative immunoblot from spinal cords of wild type, Tg*Usp14CA*, Tg*Usp14*, and *ax* ^*J*^ mice probed for ubiquitin. β**-**tubulin was used as a loading control. (B) Representative immunoblot of cortical neurons from wild type mice treated with vehicle (DMSO) or 20 μM IU1 for 24 h. (PDF 1 MB)

Additional file 4: Figure 6: No evidence of cell death in Tg*Usp14CA* spinal cord. Description of data: (A) Relative abundance of *Mkk4* mRNA in spinal cords from 4- to 6- week-old wild type and Tg*Usp14CA* mice. n = 3 animals per genotype, run in triplicate. (B) Representative immunoblots of p-p38 MAP kinase, p38 MAP kinase, pERK1/2, and ERK1/2 in spinal cords from 4- to-6 week-old wild type and Tg*Usp14CA* mice. β-tubulin was used as a loading control. (C) Quantitation of (B), pERK quantitation includes both the 42 and 44 kDa bands. (D) TUNEL staining in spinal cord sections taken from 8-week-old wild type and Tg*Usp14CA* mice. Brain slices from wild type mice were treated with DNase 1 to generate the positive control. Scale bar = 50 μm. (PDF 2 MB)

## Abbreviations

USP14: Ubiquitin-specific protease 14
NMJ: Neuromuscular junction
JNK: c-Jun N-terminal kinase
K48: Ubiquitin chain linked through internal lysine 48
K63: Ubiquitin chain linked through internal lysine 63
MLK3: Mixed lineage kinase 3
DUB: Deubiquitinating enzyme
*ax*^*J*^: USP14 deficient *ataxia* mice
*TgUsp14CA*: Transgenic mice expressing inactive USP14C114A under *Thy1.2* promoter
Tg*Usp14*: Transgenic mice expressing wild type USP14 under *Thy1.2* promoter
Ub-VME: Ubiquitin vinyl-methyl ester
MEPC: Miniature endplate current
EPC: Endplate current
AChR: Acetylcholine receptor
TA: Tibialis anterior
MKK4: MAP kinase kinase 4
SP: JNK inhibitor SP600125.
