# Supplementary figures and images for: Ubiquitin-specific protease 14 regulates c-Jun N-terminal kinase signaling at the neuromuscular junction
Source: Mol Neurodegener. 2015 Jan 10;10:3. doi: 10.1186/1750-1326-10-3 (PMC4417291; doi:10.1186/1750-1326-10-3)

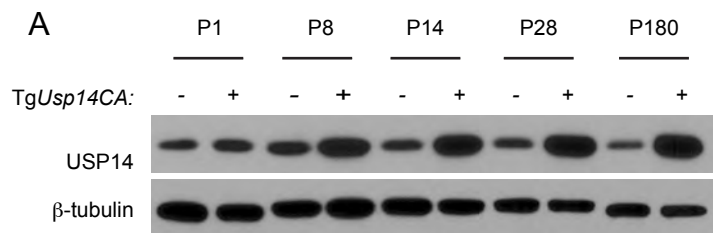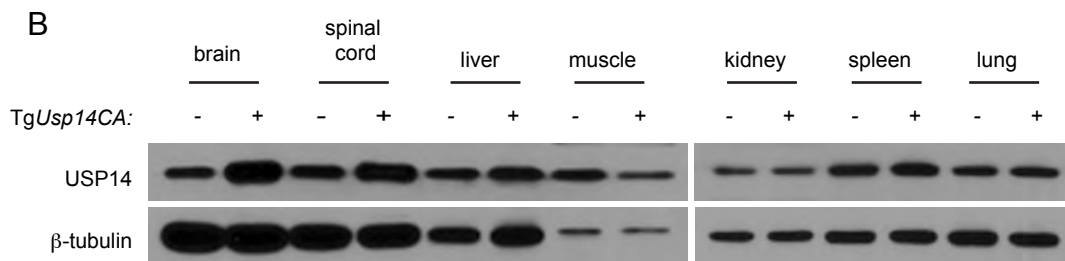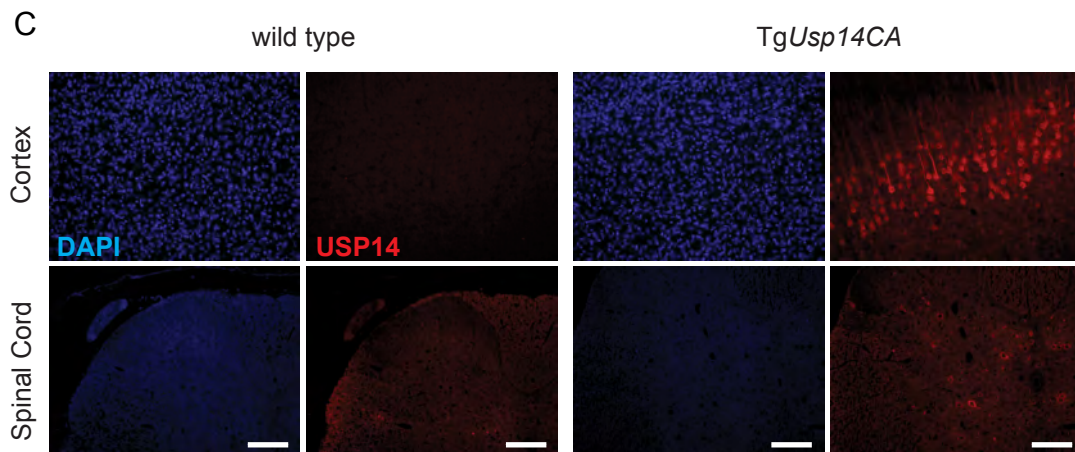

Supplement: Supplementary file 1 — Additional file 1: Figure 1: USP14CA is expressed specifically in the nervous system. Description of Data: (A) Developmental time course of USP14 expression in spinal cords of wild type (-) and TgUsp14CA (+) mice on postnatal days (P) 1-180 showing robust expression of the transgene by P8. β-tubulin was included as a loading control. (B) Representative immunoblots of USP14 from 4- to 6-week old wild type (-) and TgUsp14CA (+) mice demonstrating neuronal expression of the transgene. USP14 overexpression is assumed to reflect transgene expression. β-tubulin was included as a loading control (C) Representative immunostaining for USP14 (red) and DAPI (blue) in cerebral cortices and spinal cords of 8-week-old wild type and TgUsp14CA mice. Scale bar = 100 μm. (PDF 13 MB) [file 13024_2014_579_MOESM1_ESM.pdf]

A

wild type

*TgUsp14CA*

1 week

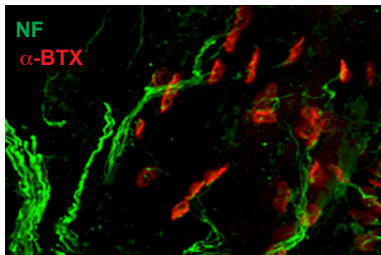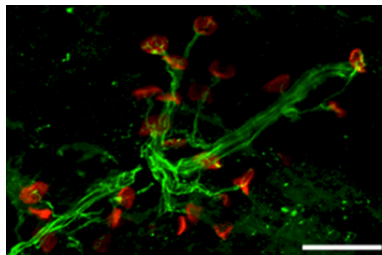

2 weeks

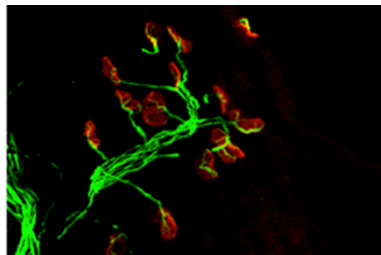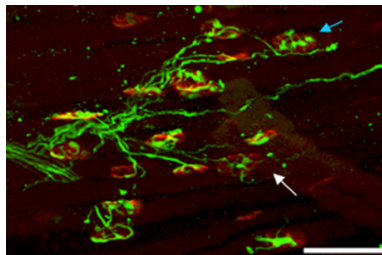

Supplement: Supplementary file 2 — Additional file 2: Figure3: TgUsp14CA mice have abnormal NMJ structure starting at 2 weeks of age. Description of data: (A) Whole-mount immunostaining of TA muscles from wild type and TgUsp14CA mice in 1- and 2-week-old. Motor neuron axons were stained with antibodies against neurofilament and synaptophysin (green), and AChRs were labeled with rhodamine-conjugated α-bungarotoxin (α-BTX, red). White arrows indicate ultra-terminal sprouting and blue arrows indicate axonal swellings, scale bars = 50 μm. (PDF 3 MB) [file 13024_2014_579_MOESM2_ESM.pdf]

A

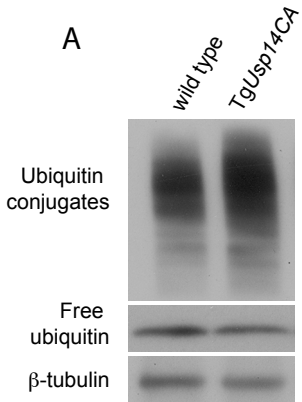

B

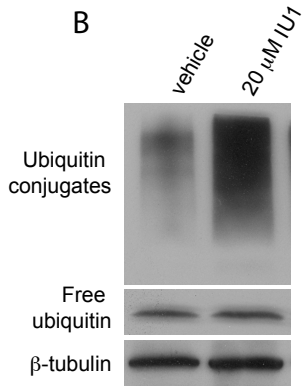

Supplement: Supplementary file 3 — Additional file 3: Figure 5: Genetic inactivation and pharmacological inhibition of USP14’s ubiquitin hydrolase activity lead to increased ubiquitin conjugates. Description of data: (A) Representative immunoblot from spinal cords of wild type, TgUsp14CA, TgUsp14, and ax J mice probed for ubiquitin. β-tubulin was used as a loading control. (B) Representative immunoblot of cortical neurons from wild type mice treated with vehicle (DMSO) or 20 μM IU1 for 24 h. (PDF 1 MB) [file 13024_2014_579_MOESM3_ESM.pdf]

A

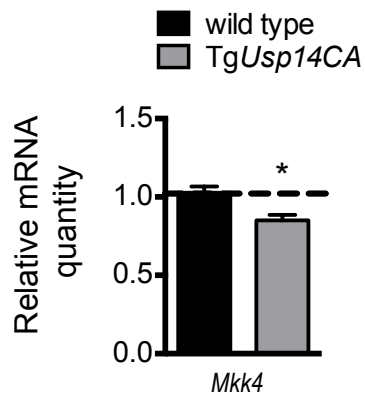

B

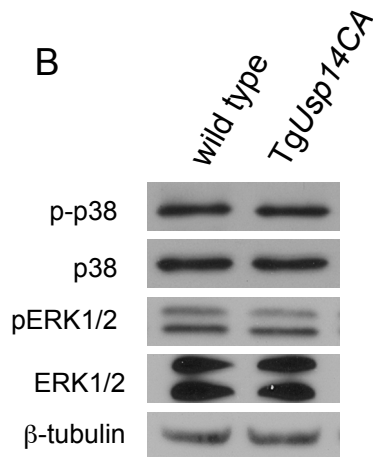

C

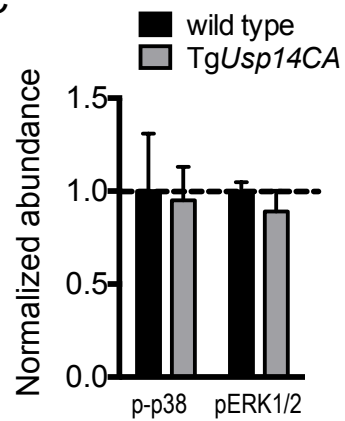

D

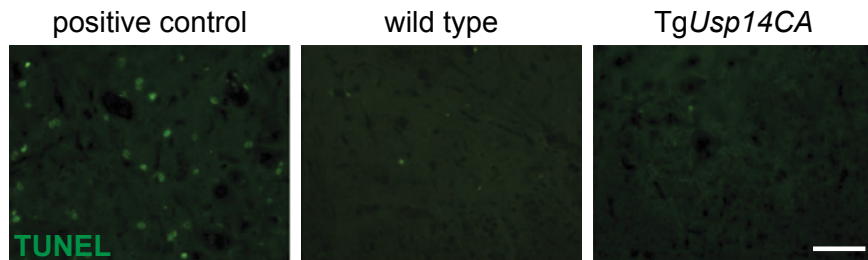

Supplement: Supplementary file 4 — Additional file 4: Figure 6: No evidence of cell death in TgUsp14CA spinal cord. Description of data: (A) Relative abundance of Mkk4 mRNA in spinal cords from 4- to 6- week-old wild type and TgUsp14CA mice. n = 3 animals per genotype, run in triplicate. (B) Representative immunoblots of p-p38 MAP kinase, p38 MAP kinase, pERK1/2, and ERK1/2 in spinal cords from 4- to-6 week-old wild type and TgUsp14CA mice. β-tubulin was used as a loading control. (C) Quantitation of (B), pERK quantitation includes both the 42 and 44 kDa bands. (D) TUNEL staining in spinal cord sections taken from 8-week-old wild type and TgUsp14CA mice. Brain slices from wild type mice were treated with DNase 1 to generate the positive control. Scale bar = 50 μm. (PDF 2 MB) [file 13024_2014_579_MOESM4_ESM.pdf]
